# Neuroinflammation in Post-Traumatic Epilepsy: Pathophysiology and Tractable Therapeutic Targets

**DOI:** 10.3390/brainsci9110318

**Published:** 2019-11-09

**Authors:** Rishabh Sharma, Wai Lam Leung, Akram Zamani, Terence J. O’Brien, Pablo M. Casillas Espinosa, Bridgette D. Semple

**Affiliations:** 1 Department of Neuroscience, Central Clinical School, Monash University, Melbourne, VIC 3004, Australia; Rishabh.Sharma@monash.edu (R.S.); Wai.Leung@monash.edu (W.L.L.); Akram.Zamani@monash.edu (A.Z.); te.obrien@alfred.org.au (T.J.O.); pablo.casillas-espinosa@monash.edu (P.M.C.E.); 2 Department of Neurology, Alfred Health, Melbourne, VIC 3004, Australia; 3 Department of Medicine (Royal Melbourne Hospital), The University of Melbourne, Parkville, VIC 3050, Australia

**Keywords:** neuroinflammation, post-traumatic epilepsy, seizures, traumatic brain injury, cytokines.

## Abstract

Epilepsy is a common chronic consequence of traumatic brain injury (TBI), contributing to increased morbidity and mortality for survivors. As post-traumatic epilepsy (PTE) is drug-resistant in at least one-third of patients, there is a clear need for novel therapeutic strategies to prevent epilepsy from developing after TBI, or to mitigate its severity. It has long been recognized that seizure activity is associated with a local immune response, characterized by the activation of microglia and astrocytes and the release of a plethora of pro-inflammatory cytokines and chemokines. More recently, increasing evidence also supports a causal role for neuroinflammation in seizure induction and propagation, acting both directly and indirectly on neurons to promote regional hyperexcitability. In this narrative review, we focus on key aspects of the neuroinflammatory response that have been implicated in epilepsy, with a particular focus on PTE. The contributions of glial cells, blood-derived leukocytes, and the blood–brain barrier will be explored, as well as pro- and anti-inflammatory mediators. While the neuroinflammatory response to TBI appears to be largely pro-epileptogenic, further research is needed to clearly demonstrate causal relationships. This research has the potential to unveil new drug targets for PTE, and identify immune-based biomarkers for improved epilepsy prediction.

## 1. Introduction

Post-traumatic epilepsy (PTE) is one of the most common long-term consequences of traumatic brain injury (TBI). The incidence of PTE varies considerably in the literature depending on injury severity, between 4% of TBI cases (reported after hospitalized mild injuries) to up to 50% (higher rates for severe TBI, in pediatric populations, and in military personnel) [1,2,3,4,5]. PTE is generally defined as recurrent and unprovoked seizures that occur at least 1 week after TBI. These are distinct from acute seizures (<1 week), which are considered to be provoked [3]. Acquired epilepsies, as opposed to epilepsies with genetic etiology, may result from a range of brain insults including stroke, infections, tumors or TBI, with PTE alone accounting for up to 20% of acquired epilepsies in the general population [3,6,7].

PTE can have a profound negative impact on an individual’s quality of life. Characterized by recurring seizures, epilepsy is a neurological disorder induced by brief and sudden disruptions of brain electrical impulses [8]. Epilepsy often presents comorbid with anxiety, depression, stigma and social isolation, and may be associated with cognitive impairments and externalizing behaviors [9]. Predicting, treating and managing PTE are all significant clinical challenges at present. While existing anti-epileptic drugs (AED) may be prescribed soon after a TBI to control the acute seizures, there is little evidence for their efficacy to prevent the development of PTE, a process known as epileptogenesis [10,11]. Further, once established, PTE is resistant to AEDs in up to 33% of patients [12,13]. Lastly, our ability to provide TBI patients with an accurate prognosis regarding their individual risk of developing PTE is hindered by an incomplete understanding of the complex pathobiology of the condition. Novel treatment strategies and predictive biomarkers, based on the fundamental neurobiological mechanisms underlying epileptogenesis after a TBI, are evidently needed.

**Figure 1:**
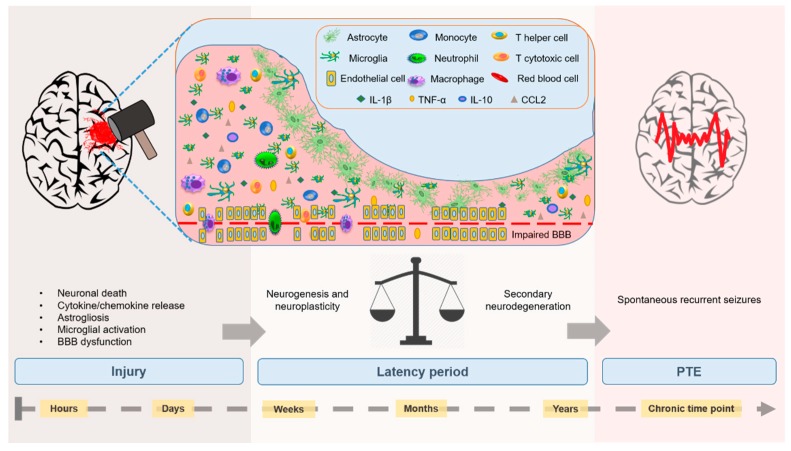
Temporal development of post-traumatic epilepsy (PTE). Traumatic brain injury (TBI) initiates a cascade of pathological processes including neuronal death and neuroinflammation, characterized by cytokine and chemokine upregulation (IL-1β, TNFα, IL-10 and CCL2), BBB dysfunction (endothelial cell disruption and red blood cell infiltration), activation of astrocytes and microglia, and infiltration of blood-derived leukocytes into the brain, including monocytes, macrophages, neutrophils and T-cells (please refer to Section 2 for further details on these factors). These processes may also influence chronic outcomes such as the development of PTE, which occurs across three phases. The first phase is the initial insult and the associated molecular and cellular mechanisms within the subsequent minutes to days. Next is a latency period (days to years after the primary injury). During this time, injury-induced neurogenesis and neuroplasticity contribute to repair and regrowth, while simultaneously, ongoing secondary injury processes promote neurodegeneration. Neuroinflammation is a key player in this response, and is thought to facilitate hyperexcitability in the brain which may ultimately result in spontaneous, recurrent seizures characteristic of PTE.

TBI is considered to be a two-phase injury: a *primary* insult, or the acute effect of external mechanical forces applied to brain; followed by a *secondary* injury phase, over the minutes, days and months post-impact [14]. Secondary injury is characterized by a robust immune response, neuronal cell death and oxidative stress, as well as augmented neurogenesis and neuroplasticity [15]. Activation of the immune response involves enhanced glial reactivity, release of pro- and anti-inflammatory immunomodulators, edema, disruption of the blood–brain barrier (BBB), and infiltration of peripheral leukocytes and lymphocytes into the injured brain. Together, these secondary injury processes augment excitatory synaptic activity while also reducing inhibitory synaptic activity, thereby altering seizure susceptibility and paving the way for epileptogenesis [16,17] (Figure 1). 

PTE may manifest, in the form of spontaneous recurrent seizures, after a variable latent period following TBI—anywhere from several months up to 10 years has been reported, with the incidence typically increasing with time [18,19]. The onset of PTE is commonly associated with hippocampal sclerosis, characterized by a loss of pyramidal neurons, activation of glial cells and enhanced excitability in the hippocampus [16,20,21]. This is strikingly similar to what is observed in sporadic patients with temporal lobe epilepsy (TLE), another common form of acquired epilepsy [20]; in fact, seizures originating from the temporal lobe have been reported in between 35% to 62% of PTE patients [22,23]. However, overlaying cortical regions have also been implicated to be involved in post-TBI epileptogenesis, with evidence of neuronal loss and neuroinflammation alongside network reorganization in the cortex after a brain injury [24,25]. 

In this narrative review, we focus on the role of neuroinflammation in the process of epileptogenesis after a TBI, drawing together evidence from both clinical studies and experimental models. The likely contributions of both innate and peripherally-derived immune cells will be considered, as well as how seizures interact with the BBB. In particular, we highlight various inflammatory mediators that are central to TBI pathophysiology, and how they have been implicated in seizures and PTE. Relevant original data manuscripts as well as reviews on the topic were sought via the PubMed database (no date restrictions), using search terms including traumatic brain injury (or brain injury) AND seizures (or epilepsy, or post-traumatic epilepsy) AND inflammation (or neuroinflammation, cytokine, chemokine, specific mediator/cell type, or process (e.g., BBB disruption, etc.). From existing reviews, where possible, original data manuscripts were sourced and referenced. Together, the current review strives towards an increased understanding of the complex interaction between inflammatory processes and neuronal excitability, with the ultimate goal being efficacious therapeutic strategies to prevent PTE development after TBI. 

## 2. Neuroinflammatory Mechanisms Driving PTE

Inflammation is a physiological process intended to protect the body from foreign invading pathogens [26]. It is closely regulated by the innate immune system, which activates and triggers the recruitment of a range of leukocytes (white blood cells) through a cascade of signals [26]. Patrolling leukocytes then detect damage- or pathogen-associated molecular patterns (DAMPs and PAMPs) expressed on the surface of foreign molecules [27]. This initiates the release of cytokines and chemokines (chemotactic cytokines), which bind to their corresponding receptors on the surface of other peripheral leukocytes to induce chemotaxis to the area of tissue damage [27]. 

More specifically, the term “neuroinflammation” refers to the inflammatory response in the central nervous system (CNS), within or associated with the brain or spinal cord. Although it was historically believed that the brain was an immune-privileged site, due to the presence of the BBB which separates the brain from the peripheral immune system, it is now widely accepted that neuroinflammation is a key component of the brain’s defense mechanisms [28]. Similar to inflammation in other body tissues, this process is regulated by cytokines, chemokines, reactive oxygen species (ROS) and other secondary messengers, which are largely produced by CNS resident and peripherally-derived inflammatory cells after a TBI [29]. In the context of TBI and secondary injury, a large body of evidence now demonstrates that neuroinflammation has opposing roles to play, contributing to both neurodegeneration as well as regeneration [30].

The hypothesis that neuroinflammation and seizure activity are intricately linked has arisen from clinical evidence of immune activation evident in the epileptic foci of brain tissue from patients with epilepsy [31,32]. It is well-established that seizures trigger the rapid activation of nearby glial cells, which respond by increasing production of inflammatory mediators to promote an inflammatory immune cascade [32,33] (Figure 1). More controversial, emerging evidence suggests that neuroinflammation may also be causal to seizures and epilepsy, by inducing changes in neuronal function and connectivity, leading to regional hyperexcitability and subsequent seizure susceptibility [15]. Much of the evidence that supports this relationship is based on the action of inflammatory cytokines and chemokines [15], as detailed in Section 2.1 and Section 2.2, and summarized in Table 1. 

### 2.1. Cytokines

#### 2.1.1. Interleukin-1 (IL-1)

IL-1 cytokines are key mediators of the inflammatory response in both focal and diffuse brain injuries [34,35,36]. By binding to the IL-1 receptor type I (IL-1RI) [37,38,39,40], IL-1 cytokines can influence a wide range of cell types in the brain; however, they may also act independently of the ligand-receptor pathway. The pro-inflammatory IL-1β is best-characterized member of the IL-1 family and is elevated in post-mortem tissue from TBI patients within minutes to hours post-trauma [41]. A robust IL-1β response has also been demonstrated in experimental models of TBI in rodents [34,40,42,43,44], whereby inhibition via antagonism of the IL-1RI, prevention of IL-1β synthesis, or genetic deletion yields neuroprotection [45,46,47]. IL-1β is an important initiator of the immune response, regulating the release of other cytokines and chemokines [48,49,50], ROS and other neurotoxic mediators to promote glial activation and proliferation [30]. Following TBI, IL-1β has been implicated in the recruitment of leukocytes [51], degradation of the extracellular matrix [52], disruption of the BBB [53], formation of edema, and even cell apoptosis [54]. 

Recently, increasing evidence suggests that IL-1β also has ictogenic properties. Clinical studies have reported elevated IL-1β in serum, CSF and brain tissue associated with a range of epileptic etiologies [31]. In vivo and in vitro laboratory experiments have further shown that IL-1β can downregulate GABA-mediated neurotransmission [55], inhibit astrocytic glutamate uptake [56], and modulate neuronal excitation via interaction with α-amino-3-hydroxy-5-methyl-4-isoxazolepropionic acid (AMPA) and N-methyl-d-aspartate (NMDA) receptors; all changes that result in excessive neuronal activity to precipitate the onset of seizures [57,58]. IL-1β has been shown to contribute to the generation of experimental febrile seizures [59], while antagonism of the IL-1RI or prevention of IL-1 synthesis is anti-seizure in TLE models, with these latter findings suggesting a role in epileptogenesis (i.e., the development of epilepsy) in addition to ictogenesis (i.e., the generation of seizures) [60,61]. Consistent with these findings, we recently demonstrated that administration of the IL-1R antagonist acutely post-TBI in young mice reduced seizure susceptibility at 2 weeks post-injury, accompanied by reduced hippocampal astrogliosis [62]. IL-1R antagonist-treated TBI mice further showed improved spatial memory and fewer evoked seizures chronically (6 months post-injury), alongside greater preservation of cortical tissue [62].

#### 2.1.2. Tumor Necrosis Factor (TNFα)

Similar to IL-1β, the effects of the cytokine TNFα are predominantly pro-inflammatory [30]. TNFα acts via a ligand-receptor pathway, with interactions of TNF with the receptor TNF-R1 (also known as p55) known to initiate apoptosis [30]. In certain cell types, however, TNFα can bind to an alternative receptor TNF-R2 (also known as p75), mediating downstream signaling pathways to regulate cell proliferation [105]. In the context of TBI, early experimental studies in which TNFα was administered or inhibited attributed detrimental effects to this cytokine, including the promotion of leukocyte infiltration, BBB degradation and neuronal degeneration [52,63,64,65,66,67]. Yet, subsequent studies using TNFα and TNF receptor knockout mice found that the lack of TNF resulted in increased neuronal loss, recovery time, and mortality rates after TBI in rodents [106,107,108]. These later studies are consistent with known restorative functions of TNFα, such as the ability to modulate neurotrophin production and protect neurons against NMDA-mediated calcium influx [68,69]. Together, the current understanding is that TNFα likely has a dual role as both a pro- and anti-inflammatory cytokine, depending upon the timing, magnitude, cellular targets and signaling cascades involved.

Similar to the dichotomous role in which TNFα plays in neuroinflammation, studies using transgenic mice have suggested that TNFα is both pro- and anti-convulsive (pro- and anti-seizure). In one study, transgenic mice with astrocytic overexpression of TNFα were observed to have shorter seizures, whereas animals lacking TNFα receptors had prolonged seizures [72]. In contrast, another study reported that transgenic mice with neuronal TNFα overexpression developed seizures and died prematurely [70]. This conflicting role of TNFα in epileptogenesis is thought to correspond to activation of the different receptors, p55 and p75. For example, Balosso and colleagues demonstrated that p75 receptor knockout mice had enhanced seizure activity, while those with a p55 receptor knockout had reduced seizure susceptibility [72]. These results suggest that the anti-convulsive activity of TNFα is mediated by the p75 pathway, while the p55 pathway is involved in the pro-convulsive activity of TNFα. However, the mechanisms which determine the predominance of either pathway over the other are still unclear, although the mediation of Ca^2+^-dependent glutamate release from astrocytes has been proposed as a means by which TNFα can promote excitatory synaptic activity in the epileptic hippocampus [71]. Further, the role of TNFα signaling in epilepsy that develops after TBI has not yet been explored. 

#### 2.1.3. Interleukin-6 (IL-6) 

The cytokine IL-6 is expressed by a number of cells in the brain, including astrocytes, microglia and neurons [109,110,111,112]. From laboratory studies, IL-6 also appears to play a conflicting role in neuroinflammation [113,114,115,116]. It can act as a pro-inflammatory cytokine to increase secretion of chemokine and adhesion molecules, and thereby enhance leukocyte recruitment [73]. On the contrary, IL-6 has been reported to inhibit TNFα production [74], reduce NMDA-mediated neurotoxicity [75], and promote neuronal differentiation and survival [76,117].

While usually undetectable in the healthy CNS, IL-6 is often the cytokine present at the highest concentration in patient CSF after a TBI [41,76,118]. Similarly, serum levels of IL-6 have been associated with a range of human epilepsy conditions [31], and it is generally regarded as being ictogenic in this context. Indeed, several patient studies have reported a correlation between elevated IL-6 levels in the CSF or plasma, with the severity of epileptic seizures [77,78,79,80,81]. This hypothesis is further supported by experimental evidence that transgenic CNS over-expression of IL-6 results in aberrant hippocampal excitation, spontaneous seizure generation and neurodegeneration [82,83,84,85].

#### 2.1.4. High Mobility Group Box Protein-1 (HMGB1) 

HMGB1 is a DNA-binding protein known to be released acutely after tissue injury as a DAMP, as well as in an active fashion by reactive inflammatory cells. By interacting with a range of receptors including RAGE (receptor for advanced glycation end-products) and TLR4, HMGB1 acts to promote further immune cell activation and release of pro-inflammatory cytokines, perpetuating the immune response [94]. 

In the CNS, HMGB1 has been implicated in cerebral edema, neurodegeneration and neuroinflammation in a range of injury paradigms and disease states, including epilepsy and TBI [98]. Indeed, increasing literature suggests that HMGB1 is involved in the initiation and propagation of seizures [98]. Elevated HMGB1 has been reported in brain tissue resected from patients with epilepsy [119], while inhibition of HMGB1 in animal models has been found to reduce seizure pathology and the development of epilepsy in acquired epilepsy models [120,121,122,123]. Several mechanisms have been proposed to explain these effects, whereby HMGB1 can act to promote neuronal hyperexcitability via NMDA receptor potentiation [96], as well as interact with IL-1β signaling pathways [15]. 

#### 2.1.5. Interleukin-10 (IL-10)

IL-10 is generally characterized as an anti-inflammatory cytokine. In combination with transforming growth factor beta (TGFβ), it inhibits a range of pro-inflammatory mediators such as IL-1α, IL-1β, IL-6, IL-8, IL-12, IL-18, TNFα and granulocyte-macrophage colony stimulating factor (GM-CSF) [86,87,88,89,90]. IL-10 has been reported to thereby regulate glial cell activation and inhibit leukocyte recruitment and accumulation, while also promoting nerve growth factor (NGF) production [91]. For example, administration of exogenous IL-10 after experimental TBI in rats was found to reduce levels of pro-inflammatory IL-1β and TNFα in the CNS [91].

An anti-seizure effect of IL-10 has also been suggested by several animal studies. For example, using rat hippocampal slices, IL-10 application was shown to reduce epileptiform activity caused by transient episodes of hypoxia [92]. Another study examining hyperthermia-induced seizures in rats, reported that the temperature needed to generate seizures in IL-10 treated rats was significantly higher than for saline-treated controls, indicating that IL-10 treatment rendered animals more resistant to seizure induction [93]. These anti-seizure effects of IL-10 are thought to be attributed to the cytokines anti-inflammatory effects [124,125]. Whether IL-10 also modulates vulnerability to seizures after a TBI has not yet been explored.

### 2.2. Chemokines

Chemokines direct the influx of immune cells to the site of injury or infection, a response that is initially protective for the host. However, persistence of immune cell recruitment and activation may lead to an over-exuberant inflammatory response, which in the context of TBI, is detrimental and promotes secondary neurodegeneration [126]. Chemokines have also been proposed to play a major role in the development of epilepsy [127]. For example, elevated levels of the chemokines CCL2, -5, -19 and -22, CXCL8 and the chemokine receptors CX3CR1 and CXCR4 are commonly reported in experimental models and patient cohorts with TBI and/or epilepsy 31, [127,128]. CCL2, -3, -4 and -5 concentrations are elevated in the hippocampus of TLE patients, and after seizure induction in experimental TLE in rats [128,129]. Other chemokine receptors such as CCR7, -8, -9 and -10 appear to be downregulated in the hippocampus in animal models of epilepsy, although the consequences of this have not yet been established [130,131]. Indeed, with the exception of CCL2 (as described in the next paragraph), there is little evidence to date to demonstrate a causal effect for chemokine signaling in epileptogenesis. 

CC Chemokine ligand 2 (CCL2), also known as monocyte chemoattractant protein-1 (MCP-1), has long been identified as a key mediator of leukocyte recruitment in the injured brain [132] (Figure 1). Produced by astrocytes within hours after injury [133], CCL2 levels are notably elevated in the brain, CSF and serum over an acute time course after TBI and associated with poor neurological outcomes [134]. CCL2 binds to its primary receptor CCR2 to recruit peripheral monocytes and promote macrophage infiltration into the injured brain 49, contributing to BBB compromise and the perpetuation of neuroinflammation [133,135,136]. CCR2 is also expressed on a range of CNS cells including microglia, astrocytes, endothelial cells and neurons [114,137,138]. 

In the context of epilepsy, CCL2 expression is robustly upregulated in human epileptic brain tissue, leading to the hypothesis that CCL2/CCR2 signaling may play a role in the pathogenesis and progression of epilepsy [31,139,140]. Several mechanisms have been explored which may account for this chemokines’ ability to enhance neuronal excitability—for example, CCL2 has been reported to modulate ion channel expression and composition 103, decrease neuronal inhibition [102], and impair synaptic currents [103]. CCL2 may also indirectly promote seizures via the induction of other pro-inflammatory cytokines such as IL-1β, which is known to promote neuronal death, neuroinflammation and PTE as described earlier [62,141,142]. While it remains unclear whether CCL2 is epileptogenic in addition to ictogenic, a recent study by Cerri and colleagues found that inference of the CCL2 signaling pathway both reduced acute lipopolysaccharide-induced seizures and prevented seizures in chronic TLE animals [104]. Further research is evidently needed, as a potential role of CCL2/CCR2 signaling in the context of PTE specifically has not yet been considered. 

### 2.3. Microglia

Microglia are specialized brain-specific macrophages which make up 10% to 20% of the glial population, with the primary purpose of immune surveillance [143,144]. Microglia act as the first form of immune defense, actively searching for infectious agents, damaged neurons and plaques in the CNS [145,146] (Figure 1). After TBI, various microglial receptors sense the presence of DAMPs and PAMPs in the extracellular space, triggering the release of cytokines and chemokines which then recruit leukocytes to the site of tissue damage [147]. Signals such as damaged cell membranes, changes in ionic and neurotransmitter levels, as well as the presence of serum proteins in the brain, further activate microglial cells and transform them into a phagocytic phenotype [148]. During this process, cytoskeletal rearrangements enhance the antigen repertoire and expression of MHC molecules on the cell surface, which assist microglia cells to migrate to the site of inflammation [29,149]. Cell activation is also associated with the release of ROS, which assists in the removal of damaged neurons [150,151,152]. Thus, microglia appear to play a neuroprotective role in response to a trauma [153]. 

However, considerable evidence also demonstrates detrimental consequences of microglial activation, particularly when they are chronically activated or primed. Production of cytotoxic molecules such as ROS and quinolinic acid contribute to oxidative stress in the injured brain, which can lead to progressive neurodegeneration [154,155,156,157]. Neuronal loss may be amplified by microglial phagocytosis of bystander cells [158]. Overactive, or persistently activated, microglia have been associated with pathological changes and cognitive deficits, attributed to the excessive production of proinflammatory mediators which trigger further inflammatory cell activation and secondary brain damage [158].

The precise roles of microglia in the epileptogenic process remains largely unclear, likely due to the complexity and heterogeneity of microglial phenotypes and responses in the brain. In the context of epilepsy, microglia and astrocyte activation is routinely observed in the epileptic brain foci from patients, alongside elevated levels of CX3CR1, IL-6 and TNF-α [159]. Similarly, changes in microglial molecular profiles and morphology are consistently detected after experimental models of epilepsy [160]. Being exquisitely sensitive to changes in the microenvironment, and major producers of pro-inflammatory mediators, activated microglia are thought to be important players in seizure onset and recurrence [161,162]. However, a recent study examined mice in which mTOR signaling was selectively elevated in microglia (Tsc1Cx3cr1 conditional KO mice), suggested that this upregulation is sufficient to induce spontaneous seizures in a manner independent of pro-inflammatory cytokine production [163].

An association between microglial activation and seizure propensity has been suggested from experimental manipulation of microglia, such as inhibition of activation with the tetracycline-derivative minocycline [164]. For example, minocycline treatment of rodents for a 2-week period following experimental status epilepticus was found to reduce the number, severity and duration of spontaneous recurrent seizures [165]. While such findings implicate microglia in epileptogenesis, other neuroprotective effects of minocycline (e.g., direct effects on neuronal activity, as suggested from in vitro experiments [166]) cannot be ruled out. Further, other studies inhibiting microglia by other means, such as pharmacological inhibition of colony-stimulating factor 1 receptor (CSF-1R), was shown to reduce microglial proliferation but did not alter the seizure response in a kainic acid mouse model [167]. Yet others have found that microglial depletion via CSF-1R administration in fact accelerated seizure occurrence and hippocampal damage; although these findings may be specific to the viral encephalitis-induced seizure model in which they were observed [168].

A direct interaction between microglia and neurons may also be an important factor in how these cells, upon activation after a TBI, contribute to epileptogenesis. Independent of cytokine release, increasing evidence suggests that microglia can influence neuronal excitability via their known roles in synaptic pruning, synaptic plasticity and neurogenesis [160]. To date, however, few studies have directly examined the role of microglia in epilepsy following TBI. 

### 2.4. Astrocytes

Similar to microglia, astrocytes also express a range of different receptors, allowing them to respond to almost all kinds of neuroactive molecules in conditions of stress or injury, such as TBI. Astrocyte activation, also known as astrogliosis, involves alterations in cell morphology and molecular profile, migration towards the site of injury, and release of cytokines and ROS [30,147,169] (Figure 1). Also similar to microglia, both neuroprotective and neurodegenerative properties have been attributed to activated astrocytes in the context of TBI. A pioneering study in the field, by Bush and colleagues in 1999, showed via experimental depletion of astrocytes that these cells promote neurite growth and are necessary for BBB repair after cortical injury in mice [170]. Astrocyte-derived neurotrophic factors and metabolites may assist in brain injury repair [171], while astrocytes are also able to uptake excess neurotransmitters to minimize excitotoxicity [171,172,173]. Conversely, over-activation of astrocytes may exaggerate the inflammatory response and cause neurodegeneration. Overabundant release of excitatory glutamate, soluble proteases, lipid mediators, cytokines, ROS and complement factors by reactive astrocytes can all promote neuronal cell death [174]. Further, formation of a glial scar, an attempt by astrocytes to isolate the injured tissue from the rest of the CNS and minimize secondary damage spread, simultaneously obstructs tissue regeneration and axon remyelination [172,173,175].

Many of these astrocytic responses to TBI could, theoretically, promote excitability of the tissue and yield a more seizure-prone microenvironment. In addition, astrocyte changes after TBI such as loss of glutamate transporter 1 (Glt1) expression [176], increased activation of mTOR signaling [177], and altered expression of K ion channels as well as neurotransmitter receptors, all provide conditions suitable for the development of PTE after a brain insult [178,179,180]. Several recent articles have reviewed the evidence that astrocytes influence vulnerability to seizures and epilepsy [181,182]. In general terms, failure to clear excess extracellular K+ leads to prolonged depolarization and hyper-excitability, leading to seizure activity. Changes in astrocyte gap junctions or altered expression, location and function of astrocyte-expressed K+ channels are evident in both clinical cases and experimental models of epilepsy [183,184]. In particular, downregulation of the co-transporter KCNJ10 (Kir4.1), an essential regulator of K+ clearance in the brain [185,186], has been linked with seizure activity after TBI [187]. Kir4.1 is also co-expressed on astrocytic endfeet along with the water channel aquaporin 4 (AQP4) [188]. As such, water transport malfunction is often evident in epileptic brain tissue. In cases of excess extracellular K+, the extracellular space is reduced, suggestive of the importance of water transport via aquaporin channels expressed on glial cells [189]. Gap junction proteins such as CX30 and CX43 may also be impaired, which result in disrupted astrocyte coupling and subsequent loss of connection between these cells. These changes in turn can lead to hyperexcitability due to excess glutamate and K+ in the extracellular space [190]. Finally, astrocyte-dependent glutamate release can trigger seizure activity, initiated by the hypersynchronous activity of neuronal potentiation [191]. Together, the above-mentioned studies (and many others as reviewed in depth elsewhere [181,182]), all point to a central role of astrocytes in epileptogenesis. 

### 2.5. Endothelial Cells and Blood-Brain Barrier Dysfunction

The BBB is a protective barrier separating the CNS tissue from the peripheral circulation. Its protective role stems from its unique structure comprised of endothelial cells, astrocyte end-feet and pericytes [192]. The endothelial cells forming the BBB are fused together via tight junctions and are encapsulated by astrocytes, obstructing the entrance of blood borne macromolecules to the brain [192]. This feature also impedes the entrance of neurotransmitters and is associated with maintaining the ionic homeostasis of the brain (as reviewed in [182,193,194,195]). Due to tight junctions present between cells, the passage of cells is limited to transcellular movement though specific channels. 

Seizure activity alone can promote prolonged BBB disruption in several ways. For example, an increase in glutamate associated with seizures can lead to elevated matrix metalloproteinase (MMP)-2 and MMP-9 levels, the enzymes that regulate integrity of the extracellular matrix. Increased MMPs may then digest the tight junction proteins and increase BBB permeability [196,197,198]. 

Early after a TBI, primary mechanical injury creates parenchymal disruption to microvessels, increasing permeability of the BBB [195] (Figure 1). This in turn allows for the entrance of serum proteins such as albumin and thrombin that are usually restricted from the brain, as well as peripherally-derived immune cells to infiltrate the brain parenchyma [199,200]. Albumin has been shown to induce proliferation of fibroblasts, resulting in changes to the astrocyte endfeet integral to the endothelial lining of BBB [193]. In a rat model of epilepsy, the extent of BBB disruption was positively correlated with an increase in seizure frequency, suggesting that BBB dysfunction is associated with the progression of epilepsy [201]. This relationship is mediated by TGFβ receptor signaling [15,202]. An increase in micropinocytosis and disruption of tight junctions is also a common consequence of brain injury, which has been shown to result in BBB permeability, hyper-excitability and seizure susceptibility in epileptic tissue [203]. Of note, while increased BBB permeability as a result of TBI can resolve within weeks, dysfunction may persist for years in patients that develop PTE [204,205]. This suggests that the mechanisms leading to BBB disruption and associated PTE after TBI are dependent on the post-injury phase; and that understanding the temporal changes in the relationships and activities of glia and neurons may shed light on novel anti-epileptic strategies across this time course. 

### 2.6. Blood-Derived Leukocytes

In addition to the activation of CNS resident immune cells, microglia and astrocytes, peripheral blood-derived leukocytes including neutrophils, monocytes/macrophages and T cells can infiltrate the CNS in both TBI and epilepsy disease states. Leukocytes gain passage into the CNS as a consequence of BBB damage or dysfunction, as well as via active recruitment in response to chemokine signaling [144,149,150,151]. 

#### 2.6.1. Neutrophils

Neutrophils are among the first cellular responders to a TBI from outside the CNS, with active recruitment into the brain parenchyma evident with the first few hours [106,206,207,208]. In comparison to the dual roles that astrocytes and microglia serve in the injured brain, most evidence concurs that infiltrated neutrophils are predominantly detrimental to the CNS. Activated neutrophils release a range of enzymes and proteases that disrupt microvessels, alter cell membranes, and promote BBB degradation [153,209,210]. Neutrophils are also a significant producer of ROS, promoting oxidative stress and subsequent neurodegeneration after TBI [153,211]. Experimental data suggests that, even in the absence of an injury, the presence of neutrophils in the brain contributes to considerable neuronal loss, BBB breakdown and glial cell abnormalities [211].

In patients with epilepsy, higher numbers of neutrophils in the CNS have been detected compared to control patients without epilepsy [212], consistent with what is observed in animal models of epilepsy [213]. The pro-inflammatory microenvironment including the presence of IL-1β, TNF-α, CXCL-1, -2, and -5, released by other immune cells in the injured or epileptic brain, promotes further neutrophil activation and activity, augmenting oxidative damage and BBB permeability [214,215]. As described earlier, these neuropathological changes may promote the progression of epileptogenesis. To our knowledge, no studies to date have considered the effect of neutrophil manipulations (e.g. neutrophil depletion experiments) on seizure outcomes after acquired brain injuries, which would provide insight into what contribution these cells may have relative to other activated immune cells in this context.

#### 2.6.2. T-Cells 

Neutrophils number have been shown to increase in the circulation as well as the CNS after a TBI [216], where they can influence cells of the adaptive immune system including CD8+ T cells, regulatory T cells, and CD4+ T helper cells [217]. Although to a lesser extent compared to neutrophils and monocytes/macrophages, T cells also infiltrate the injured and epileptic brain, where they contribute to ongoing neuropathology and disease severity [218,219,220]. An important role for peripheral adaptive immune cells has been suggested by experimental depletion of regulatory T cells in an epilepsy model, which resulted in increased seizures [219]. Taken together, findings to date suggest that adaptive immunity acts to delay or prevent seizure onset, which could potentially be employed as a novel therapeutic means to control epileptogenesis [221,222].

#### 2.6.3. Monocytes and Macrophages 

Blood-derived monocytes and macrophages may infiltrate the acutely injured brain via BBB disruption, but are also actively recruited via chemotaxis via a multi-step process of transmigration and infiltration into the brain after TBI [223,224]. Monocyte-derived macrophages are present in large numbers in the damaged tissue by 3 to 5 days after a TBI, where they appear to be more detrimental than beneficial in purpose [30]. Specifically, upon differentiation to macrophages, these cells engage in phagocytosis, release of pro-inflammatory cytokines and chemokines, and antigen presentation; while also promoting axonal regrowth and wound healing [225,226]. Consistent with evidence that peripherally-derived macrophages contribute to ongoing neurodegeneration and secondary brain damage, experimental reduction of monocyte numbers by depletion, genetic manipulation or targeting CCL2/CCR2 chemokine signaling, is neuroprotective after TBI [133,225,227,228]. 

Defining a role for infiltrated macrophages in epileptogenesis has been challenging to date. CCR2+ macrophage infiltration has been observed in epileptic tissues from both patients and experimental models, suggesting an association between cell accumulation and epileptiform activity [222]. Supporting a causal relationship, experiments to pharmacologically manipulate macrophage infiltration via inhibition of CCL2 or CCR2 has been shown to suppress lipopolysaccharide-induced seizures in mice [104]. However, recent experimental studies have suggested that these cells contribute to neurodegeneration after TLE, independent of any effect on seizure vulnerability [222,229,230]. Further work to characterize how these cells function and change over time after TBI, during the latency period when epileptogenesis is ongoing, may provide greater insight into the relationship between macrophages and epilepsy. 

## 3. Inflammatory Mediators as Biomarkers of PTE

Together, the current body of evidence suggests that a wide range of immune cell types and their secreted cytokines and chemokines can influence not only the inflammatory response to TBI, but also contribute to seizure generation and the process of epileptogenesis, driving the brain towards a hyperexcitable state that is more vulnerable to PTE. While further research is evidently needed to demonstrate a causal relationship for most of these cells and factors, the evidence nonetheless supports the concept of neuroinflammatory processes serving as potential biomarkers of PTE. Biomarkers are urgently needed in this context, to better predict which individuals after a TBI are at greatest risk of developing PTE, as well as assist with improved clinical trial design for the investigation of novel disease-modifying therapies [231]. 

Probably the best-characterized immune-related protein biomarker to date in the context of TBI is glial fibrillary acidic protein (GFAP). Released by damaged and/or dying astrocytes, serum and CSF levels of GFAP are considered a reasonable indicator of brain injury severity [231]. Specific to PTE development, only a handful of studies to date have considered the utility of inflammatory cytokines as biomarkers. For example, Diamond and colleagues (2014) found that CSF/serum IL-1β ratios in moderate to severe TBI patients were associated with PTE risk [232]. Further, polymorphisms of the IL-1 gene were associated with serum levels and differential PTE risk, suggesting that a genetic predisposition may also play an important role in an individuals’ vulnerability to epilepsy following TBI [232]. Others have suggested that the robust elevation of other inflammatory mediators such as IL-6 or CCL2, in CSF and serum acutely after TBI or first seizure, may be useful to predict the progression from first ictal event to PTE; however, these have not yet been validated [31]. Another avenue that is being explored for epilepsy prediction is positron emission tomography (PET) imaging for the translocator protein TSPO, an outer mitochondrial membrane protein expressed by microglia, as a non-invasive indicator of glial inflammation preceding acquired epilepsy [233,234,235,236]. 

## 4. Inflammation as an Anti-Epileptic Therapeutic Target 

As discussed above, the activation of immune signaling pathways in neurons and glia results in the pathological modification of cell function, synaptic transmission and plasticity. Ultimately, these changes promote the development of neuronal hyperexcitability underlying seizure generation, cell loss, and epilepsy comorbidities [237]. Despite an increasing awareness of neuroinflammation as a critical mechanism in epileptogenesis, the potential anti-epileptogenic effect of immune-targeting pharmaceuticals have been minimally explored in PTE patient populations to date [238]. However, targeted anti-inflammatory compounds have recently been explored in preclinical models of PTE for antiepileptogenic and disease-modifying properties. 

In the context of PTE, the IL-1R1 antagonist Anakinra was shown to reduce long-term seizure susceptibility and to modulate the astrocytic response and in a mouse model of pediatric TBI [62]. In contrast, while the modulation of HMGB1 has been postulated to have anti-ictogenic activity in experimental epilepsy models, as described earlier, inhibition via glycyrrhizin in the same TBI model did not alter seizure outcomes [239]. Of note, there is likely considerable cross-talk between inflammatory mediators—for example, HMGB1 can enhance expression of IL-1β, while IL-1β can promote HMGB1 translocation from the nucleus to the cytoplasm in preparation for its release [240]. In light of these findings, a combination of anti-inflammatory drugs may be a more promising antiepileptogenic strategy [237]. In rats, a multi-pronged approach of targeting IL-1R1 with Anakinra, HMGB1 with the pseudo-peptide inhibitor Box A, and the NMDA receptor with ifenprodil, has been shown to delay the development of spontaneous seizure onset and disease progression. Similarly, in a mouse model of acquired epilepsy, combined therapy of VX-765, a caspase-1/ IL-1β synthesis inhibitor, and the TLR4 antagonist Cyanobacterial LPS, reduced the spontaneous seizures and improved memory deficits in epileptic animals [120]. 

The apparent importance of TGF-β signaling and BBB integrity has also generated interest as a therapeutic target for epilepsy development after TBI [238]. For example, in a model of focal neocortical BBB disruption, targeting the signaling cascade downstream of albumin-TGFβ activation was found to effectively prevent epilepsy [241]. Treatment with losartan, an angiotensin II type 1 receptor antagonist with TGF-β signaling effects, has been shown to reduce the development and severity of seizures, alongside neuroinflammation, in rodent models of acquired epilepsy [242,243]. Losartan has been shown to improve cognition after experimental TBI [244], but its effects on epilepsy following TBI have not been examined. 

Proof-of-concept clinical trials and case report studies in different types of epilepsy have reported promising disease-modifying efficacy of clinically approved target-specific anti-inflammatory drugs. A phase IIA randomized, double-blind, placebo-controlled trial in patients with drug-resistant focal-onset receiving VX-765 reported seizure reduction in some patients, that persisted for a period of time after drug discontinuation [245]. Case reports of Anakinra use for epilepsy of differing etiologies have also suggested efficacy at improving seizure control in some patients [246,247]. Similarly, Adalimumab, a monoclonal anti-TNFα antibody, yields a reduction in seizures in some patients with Rasmussen’s encephalitis [248]. More generally, the broad-spectrum anti-inflammatory antibiotic minocycline was reported to reduce seizure frequency in a patient with astrocytoma and drug-resistant epilepsy [249].

It is also worth noting that some common medical interventions prescribed to patients with epilepsy, such as anti-inflammatory non-steroidal drugs, steroids, cannabinoids, or a ketogenic diet, have been shown to display anti-inflammatory properties, which may mediate some of their therapeutic effects [237]. However, there is a current lack of systematic controlled studies to clearly delineate this relationship. 

Regardless, this encouraging early evidence suggesting benefit from pharmacological interventions targeting inflammatory response in clinical and experimental epilepsy warrants further investigation. Novel antiepileptogenic targets may be identified within the complexity of the neuroinflammatory response to a TBI. Similarly, repurposing drugs already approved for other medical conditions may facilitate the clinical translation of experimental findings. 

## 5. Conclusions

Neuroinflammation and neuronal hyperexcitability have long been recognized to coincide. More recently, evidence has begun to emerge to support immune activation as a mechanistic player in epilepsy etiology, rather than purely reactive response to seizure activity. A range of pro- and anti-inflammatory mediators, released from activated microglia, astrocytes and blood-derived leukocytes, have been shown to also have ictogenic or anti-seizure properties in animal models of acquired epilepsy. However, there is a notable lack of conclusive evidence for a causal role of neuroinflammatory processes in epileptogenesis, particularly after a TBI, such that future research is needed. As neuroinflammation is a common response to TBI, it also remains unclear why some patients develop PTE while others do not. This suggests that the pro-seizure consequences of immune activation in this context are complex, likely to depend upon the nature of the insult, magnitude of response, and interactions between neuroinflammation and other secondary injury cascades to consequently modulate neuronal excitability. An improved understanding of how inflammation contributes to epileptogenesis after TBI is key to the development of immune-targeted therapies, aiming to alleviate secondary damage as well as prevent PTE development after TBI. 

## Figures and Tables

**Table 1:** Key cytokines and chemokines: evidence of pro- and anti-inflammatory properties after TBI and implications for seizures/epilepsy.

Mediator	Pro-Inflammatory	Anti-Inflammatory	Role in Seizures/Epilepsy?
**IL-1β**	Regulates release of cytokines, chemokines, ROS, and proteases [48,49,50,52]. Mediates leukocyte recruitment [51], BBB disruption [53], edema [54], cell apoptosis [54] and glial activation [30]	-	IL-1β: pro-ictogenic [56,57,58,59] IL-1R: anti-seizure and anti-epileptogenic [60,61,62] (experimental and clinical evidence)
**TNFα**	Mediates leukocyte infiltration, BBB disruption, and neuronal degeneration [52,63,64,65,66,67]	Modulates neurotrophin production; protect neutrons against NMDA-mediated calcium influx [68,69]	Pro-ictogenic [70,71] Anti-seizure [72] (receptor-dependent effects) (experimental evidence)
**IL-6**	Increases secretion of chemokines and adhesion molecules, to enhance leukocyte recruitment [73]	Inhibits TNFα production and reduces NMDA-mediated toxicity [74,75]. Induces synthesis of NGF [76]	Pro-ictogenic [77,78,79,80,81,82,83,84,85] (experimental evidence)
**IL-10**	-	Inhibits cytokine production [86,87,88,89,90]. Regulates glial activation, inhibits macrophage accumulation, and promotes NGF production [91]	Anti-seizure [92,93] (experimental evidence)
**HMGB1**	Released passively by necrotic cells, or actively by immune cells; promotes inflammatory cytokine release [94,95]	-	Pro-ictogenic [96,97,98] (experimental and clinical evidence)
**CCL2**	Release by infiltrated macrophages further promotes peripheral macrophage infiltration [48]	Absence of CCL2 results in delayed secretion of different pro-inflammatory cytokines [99,100]	Pro-ictogenic [101,102,103,104] (experimental evidence)

Table abbreviations: Blood-brain barrier (BBB); CC chemokine ligand 2 (CCL2), high mobility group box protein-1 (HMGB1), interleukin-1 (IL-1), nerve growth factor (NGF), N-methyl-D-aspartate (NMDA), reactive oxygen species (ROS), tumor necrosis factor alpha (TNFα).
